# Animal model for high consumption and preference of ethanol and its interplay with high sugar and butter diet, behavior, and neuroimmune system

**DOI:** 10.3389/fnut.2023.1141655

**Published:** 2023-03-30

**Authors:** Renato Elias Moreira-Júnior, Mauro Andrade de Freitas Guimarães, Miguel Etcheverria da Silva, Tatiani Uceli Maioli, Ana Maria Caetano Faria, Ana Lúcia Brunialti-Godard

**Affiliations:** ^1^Laboratório de Genética Animal e Humana, Departamento de Genética, Ecologia e Evolução, Instituto de Ciências Biológicas, Universidade Federal de Minas Gerais, Belo Horizonte, Brazil; ^2^Laboratório de Imunobiologia, Departamento de Bioquímica e Imunologia, Instituto de Ciências Biológicas, Universidade Federal de Minas Gerais, Belo Horizonte, Brazil

**Keywords:** alcohol preference, reward system genes, neuroinflammation, behavior, high sugar and butter diet

## Abstract

**Introduction:**

Mechanisms that dictate the preference for ethanol and its addiction are not only restricted to the central nervous system (CNS). An increasing body of evidence has suggested that abusive ethanol consumption directly affects the immune system, which in turn interacts with the CNS, triggering neuronal responses and changes, resulting in dependence on the drug. It is known that neuroinflammation and greater immune system reactivity are observed in behavioral disorders and that these can regulate gene transcription. However, there is little information about these findings of the transcriptional profile of reward system genes in high consumption and alcohol preference. In this regard, there is a belief that, in the striatum, an integrating region of the brain reward system, the interaction of the immune response and the transcriptional profile of the Lrrk2 gene that is associated with loss of control and addiction to ethanol may influence the alcohol consumption and preference. Given this information, this study aimed to assess whether problematic alcohol consumption affects the transcriptional profile of the *Lrrk2* gene, neuroinflammation, and behavior and whether these changes are interconnected.

**Methods:**

An animal model developed by our research group has been used in which male C57BL/6 mice and knockouts for the Il6 and Nfat genes were subjected to a protocol of high fat and sugar diet intake and free choice of ethanol in the following stages: Stage 1 (T1)—Dietary treatment, for 8 weeks, in which the animals receive high-calorie diet, High Sugar and Butter (HSB group), or standard diet, American Institute of Nutrition 93-Growth (AIN93G group); and Stage 2 (T2)—Ethanol consumption, in which the animals are submitted, for 4 weeks, to alcohol within the free choice paradigm, being each of them divided into 10 groups, four groups continued with the same diet and in the other six the HSB diet is substituted by the AIN93G diet. Five groups had access to only water, while the five others had a free choice between water and a 10% ethanol solution. The weight of the animals was evaluated weekly and the consumption of water and ethanol daily. At the end of the 12-week experiment, anxiety-like behavior was evaluated by the light/dark box test; compulsive-like behavior by Marble burying, transcriptional regulation of genes *Lrrk2*, *Tlr4*, *Nfat*, *Drd1*, *Drd2*, *Il6*, *Il1β*, *Il10*, and *iNOS* by RT-qPCR; and inflammatory markers by flow cytometry. Animals that the diet was replaced had an ethanol high preference and consumption.

**Results and discussion:**

We observed that high consumption and preference for ethanol resulted in (1) elevation of inflammatory cells in the brain, (2) upregulation of genes associated with cytokines (*Il6* and *Il1β*) and pro-inflammatory signals (iNOS and Nfat), downregulation of anti-inflammatory cytokine (Il10), dopamine receptor (Drd2), and the *Lrrk2* gene in the striatum, and (3) behavioral changes such as decreased anxiety-like behavior, and increased compulsive-like behavior. Our findings suggest that interactions between the immune system, behavior, and transcriptional profile of the *Lrrk2* gene influence the ethanol preferential and abusive consumption.

## Introduction

Alcohol Use Disorder (AUD) is a multifactorial condition characterized by compulsive consumption, in which the genetic component is an important risk factor, with heritability estimated at around 55% (1–3). The problematic use of this drug is responsible for approximately 3.3 million deaths per year and is associated with disease development such as gastritis, hepatitis, cirrhosis, heart disease, anorexia, infections, cancer, Parkinson’s, anxiety disorders, and dementia (4–9). Studies on neurobiology and AUD causes play a key role in the investigation of explanations and therapies for this pathology.

A typical characteristic of alcohol use is behavioral alterations (9–12). Alcohol is associated with many risk behaviors, such as violent acts, self-harm, mood instability, decision-making difficulties, attention, and memory problems. Furthermore, its abstinence results in tachycardia, insomnia, hallucinations, depression, and anxiety (13–15). Indeed, Pascual et al. (16) found that in an animal model that underwent self-administration of ethanol for 5 months, just 1 day of drug withdrawal was enough to induce anxiety in the animals. Moreover, in this study, it was demonstrated that the activation of the innate immune system can influence ethanol seeking behavior (16).

There is growing evidence to suggest the activation of the immune response and inflammation in behavior disorders, as well as in the problematic use of ethanol and associated brain damage (17–21). In this scenario, it is known that beyond contributing to neurodegeneration, inflammatory signaling is also associated with alcohol dependence, since after drinking alcohol use, infiltrating macrophages and microglia become activated and induce the release of pro-inflammatory cytokines such as IL-6 and IL1β that result in neuroinflammation and blood–brain barrier breakdown (22–25). Additionally, toll-like innate immune receptor 4 (TLR4) activation in the brain during chronic ethanol abuse also triggers the production of cytokines and various inflammatory mediators (18, 26). TLR4 KO mice do not show induction of cytokines and chemokines due to ethanol use, or behavioral differences observed during abstinence (16, 27). Notably, the inflammatory process is observed in the striatum, changing its function and stimulating the drug compulsive use, even in the presence of negative consequences (16). Changes in the striatum are associated with compulsion as this brain region plays a central role in goal-directed behaviors and it is part of the Mesolimbic Dopaminergic System, popularly known as the reward system (28–30).

In this context, our research group investigated the relationship between gene transcription in the striatum and ethanol inflexible intake, defined by high preference even after adulteration of the substance (31–33). In our results, we observed several genes differentially transcribed in the LRRK2 pathway, including the main one in this pathway, *Lrrk2*. In our results, we observed several genes differentially transcribed in the LRRK2 pathway, including *Lrrk2* the main one in this pathway (31, 34–38). This gene produces an kinase anchoring protein (AKAP) that modulates the activity of protein kinase A (PKA), which is involved in the regulation of dopamine receptors (*Drd1* and *Drd2*) transcripts in neurons that project to the striatum in the reward system leading to ethanol preference (31, 34–38). In addition, it is known that the *Lrrk2* gene is related to the immune system and is biochemically associated with molecular pathways that regulate inflammation, autophagy, and phagocytosis. In this sense, *Lrrk2* polymorphisms have already been associated with inflammatory diseases such as inflammatory bowel disease, tuberculosis, and leprosy (39, 40).

LRRK2 knockdown animal models or the inhibition of its kinase activity in microglia have already been shown to decrease the production of pro-inflammatory cytokines and the hyperexpression of LRRK2 protein seems to exacerbate brain neuroinflammation by increasing its kinase activity (41–44). Additionally, the *Lrrk2* gene has a special relationship with TLR4, which, when activated by lipopolysaccharides (LPS), signals through the adapter protein MyD88 (Myeloid differentiation primary response 88) (45, 46). This may influence, in a still unknown way, its subcellular localization, overexpression, and production of inflammatory cytokines (45, 46). On the other hand, Lrrk2 is also associated with the nuclear factor of activated T-cells (NFAT), responsible to produce inflammatory cytokines *via* calcium signaling, thus being an important mediator of the immune response (46–48). NFAT signaling is inhibited by NFAT repressor non-coding RNA (NRON), a complex composed of 11 proteins, five of which are associated with LRRK2 (48, 49). LRRK2 is reported to be a negative regulator of NFAT that can inactivate its function and block its response (45, 47, 50). It is known that the LRRK2 protein is widely expressed in the brain, and that data suggests that it may play distinct roles according to its cellular sublocation and the process in which it is acting (39). Therefore, the importance of the *Lrrk2* gene in the cerebral immune system cannot be denied. However, in the AUD context, more studies are needed to understand its action on the striatum concerning the immune system and reward system.

Although ethanol has important effects on the organism, most of the studies conducted so far have focused on the neurobiology of alcoholism, showing the influence of ethanol consumption on brain circuits related to decision-making and reward processing (1, 51). Few studies are performed on the molecular regulation of control-related genes of the reward system in alcohol consumption and its association with the immune system and inflammation, such as cytokines IL-6, IL1β, and IL-10, and signals such as iNOS and NFAT. It is then hypothesized that there is an interaction between ethanol preference, the *Lrrk2* gene, and the innate immune response in the striatum. In an animal model with a sugar and butter-rich diet and ethanol intake developed by our research group, we observed that when withdrawing the HSB diet and exposing the mice to the paradigm of free choice of ethanol, there is an increased intake and preference for a drink (52, 53). In the study, we raised the possibility that this increase is being directed *via* transcriptional regulation of dopamine receptors (52). Considering the relationship between these receptors and the *Lrrk2* gene and the latter with the immune response, it is plausible to consider the possibility that this interaction is associated with high ethanol consumption and preference in this model. Thus, the present study aims to evaluate the relationship between *Lrrk2* and the immune system and how this is associated with alcohol intake and preference in a model of consumption of a HSB diet and free choice of ethanol. In this context, for a better understanding of the role of the immune system in ethanol intake, we applied the model described above, also in animal knockout (KO) for the cytokine IL6 and the transcription factor NFAT.

## Methodology

### Animals

Sixty male C57BL/6 mice, specific pathogen-free (SPF), were provided by the Animal facility of Universidade Federal de Minas Gerais (UFMG) at 6 weeks of age. Another 40, 20 C57BL/6 *Il6* KO and 20 C57CLBL/6 *Nfat* KO males were provided by the Laboratory of Immunology of Infectious Diseases at UFMG. Only male mice were used to avoid interference from the hormonal fluctuation present in females and behavioral changes of males in their presence. During the 12-week experiment, the animals were individualized in mini-isolators housed in a ventilated rack (ALESCO, São Paulo, Brazil) with a 12-h light/dark cycle. They had free access to diet, water, and/or a 10% ethanol (EtOH) solution, according to the experimental design. This study was approved by the ethics committee of the university (CEUA-UFMG; protocol number: 73/2021). Every effort was made to ensure animal welfare.

### Experimental design

**Figure 1:**
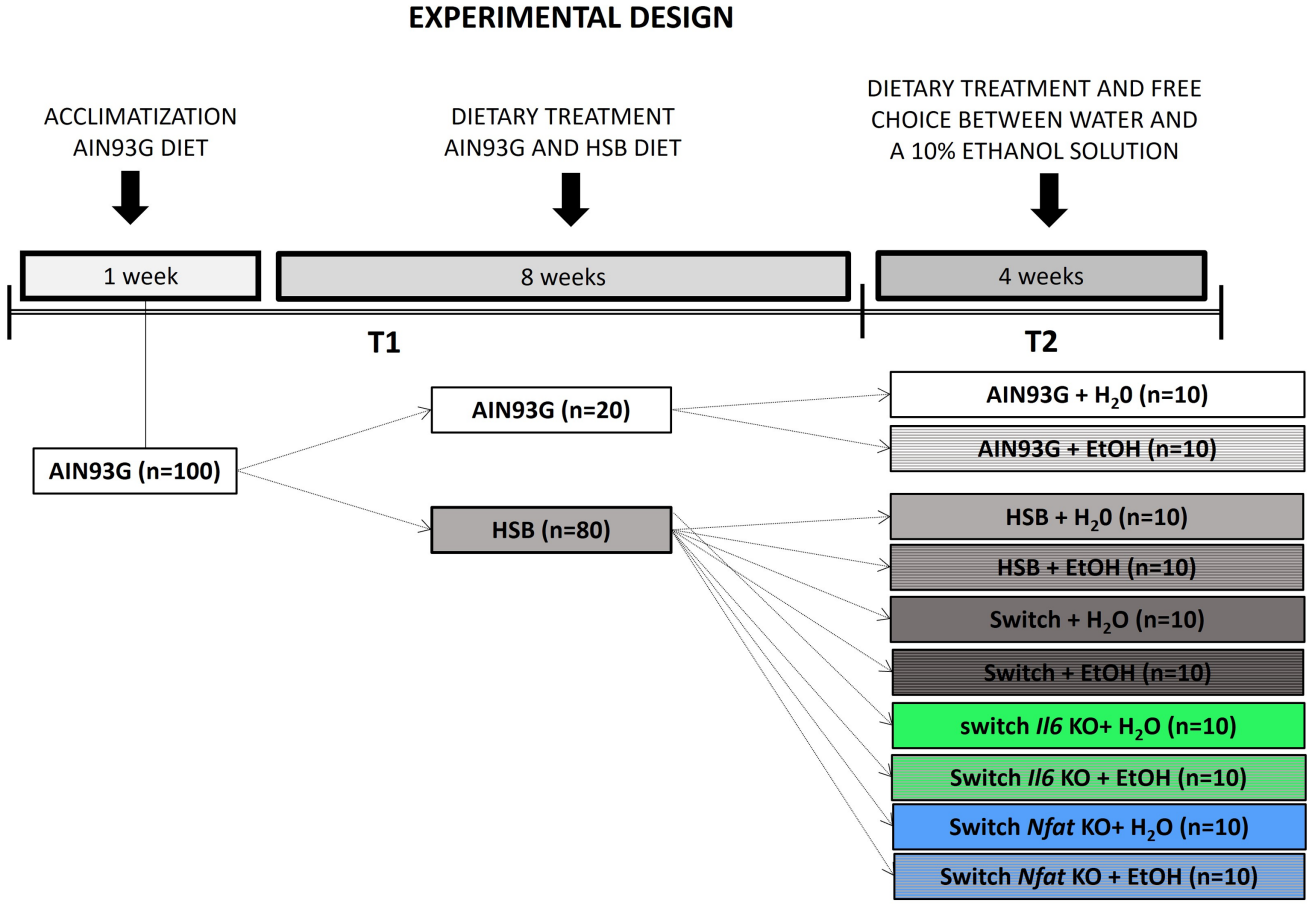
Experimental design. First, the animals underwent a one-week acclimation period and were fed the standard *American Institute of Nutrition 93-Growth* (AIN93G) diet. At the next 8 weeks (stage T1) the mice were divided into two groups: one group was fed the standard AIN93G diet (*n* = 20) and the other was fed a high sugar and high fat (HSB) diet (*n* = 80). In stage T2, lasting a total of 4 weeks, the animals were subdivided into 10 groups, named according to their particular treatments: AIN93G + H_2_O (*n* = 10), AIN93G + EtOH (*n* = 10), HSB + H_2_O (*n* = 10), HSB + EtOH (*n* = 10), Switch + EtOH (*n* = 10), Switch + H_2_O (*n* = 10), Switch *Il6* KO + H_2_O (*n* = 10), Switch *Il6* KO + EtOH (*n* = 10), Switch *Nfat* KO + H_2_O (*n* = 10) e Switch *Nfat* KO + EtOH (*n* = 10). During the 4-week T2 period, five groups (+H_2_O) had access to water only, while the remaining five (+EtOH) had free choice between water and a 10% ethanol solution. During T2, in the Switch groups, the HSB diet was replaced by the AIN93G diet.

The experiment was performed in two steps, according to the protocol described in detail in (52, 53). The first stage (T1) lasted 8 weeks, in which the mice were randomly divided into two groups: those fed the American Institute of Nutrition 93-Growth (AIN93G) control diet (*n* = 20) and those fed the High Sugar and Butter (HSB) diet (*n* = 80) (54, 55). The groups were named according to the diet consumed: AIN93G and HSB. The second stage (T2) lasted 4 weeks, in which the animals were subdivided into 10 groups, named according to their specific treatment and genetic status: AIN93G + H_2_O (*n* = 10), AIN93G + EtOH (*n* = 10), HSB + H_2_O (*n* = 10), HSB + EtOH (*n* = 10), Switch + H_2_O (*n* = 10), Switch + EtOH (*n* = 10), Switch *Il6* KO + H_2_O (*n* = 10), Switch *Il6* KO + EtOH (*n* = 10), Switch *Nfat* KO + H_2_O (*n* = 10), and e Switch *Nfat* KO + EtOH (*n* = 10). During this period, five groups (+H_2_O) had access to water only, while the other five (+EtOH) had free choice between water and a 10% ethanol solution. In the Switch groups the HSB diet was switched to the AIN93G diet when starting T2. Figure 1 illustrates the experimental design.

Throughout the experimental protocol, the body weight of the mice was measured weekly. During T2, ethanol and water intake was observed daily. Specimens were euthanized 1 day after the end of T2 during the light cycle. At euthanasia, perigonadal adipose tissue and spleen were collected from all mice. The perigonadal adipose tissue was washed with saline solution and weighed for determination of the adiposity index, according to (56). In addition, the brains of 40 animals (four from each group) were collected for flow cytometry, and, from the other 60 ones, the striatum was extracted and used for molecular analyses.

### Consumption and preference for ethanol

Measurements were made according to (52, 53). The daily consumption of water and alcohol was established by subtracting the initial weight by the final weight of the bottles (in grams) and then divided by the weight of the animal for the week. The bottles with 10% ethanol had their liquids changed after each measurement to counteract the loss of the substance by evaporation. Preference was determined when the percentage of alcohol ingested in relation to the total liquid consumed constituted a value statistically greater than the hypothetical threshold of 50.1% (52).

### Behavioral tests

The behavior of the animals was observed from the Marble burying and the light/dark box tests. On the penultimate day of T2, the specimens were subjected to the Marble burying test, which is classically used for the purpose of investigating impulsive and obsessive–compulsive like behaviors (57–59). The test and its analysis were performed according to (52, 60). In this process, each mouse was placed individually in a standard cage lined with five centimeters of sawdust. Eighteen marbles were arranged in three rows of six, evenly distributed. The animals were assessed for their burrowing behavior after 10 min, when the number of marbles with at least 2/3 of their size covered by sawdust were counted. This counting was conducted by two independent researchers, and an average number of balls buried was then established, which was used to measure the obsessive–compulsive behavior of each animal (57).

The light/dark box test was performed as described by (61). This test, based on mice natural aversion to open, brightly lit places and the spontaneous exploratory behavior of rodents in response to mild stressors, is intended to study anxiety-like behavior (62). Initially, on the last day of T2, each specimen was allocated to the dark compartment of the box, which has passages to allow free transit between this area and the light one. The mice were filmed for 5 min while they explored these sections and the videos were observed in EthoVision® XT version 12 software (Noldus Information Technology, Utrecht, The Netherlands) (63). The time spent in the clear compartment, latency, number of transitions, and the distance moved in the clear part were recorded. In this scenario, shorter time in the light compartment and longer latency is associated with anxiety-like behavior in animals (62, 64).

### Leukocyte extraction and flow cytometry

The extraction of leukocytes from the brain was adapted from (65). Once collected, the brains were macerated and kept in Falcon tubes (50 mL) with 4 mL of DMEM medium supplemented with collagenase D at 250 μg/mL in a CO2 stove at 37°C for 45 min. Then 10 mL of DMEM with EDTA (2 Mm) was added and the samples were centrifuged for 5 min at 450 *g*, 4°C. The supernatant was discarded, and the cells were resuspended in 37% Percoll. This suspension was added to another Falcon tube (15 mL) containing 3 mL of 60% Percoll and centrifuged at 950 *g* at 24°C for 20 min. After centrifugation, the ring of mononuclear cells was collected, transferred to Falcon tubes (15 mL) with 10 mL of complete DMEM and centrifuged again at 450 *g* for 5 min. Finally, the samples were resuspended in 200 μL of PBS with 0.2% fetal bovine serum. In spleen extraction, the cell suspensions were homogenized and centrifuged for 10 min at 1,200 rpm, 4°C. Lysis of red blood cells from the spleen was performed with 10X PBS diluted 1:10 in water. Subsequently, the cells were centrifuged again and resuspended in RPMI 1640 (GIBCO BRL) plus 10% fetal bovine serum, 2 mM L-glutamine, 20 μg/mL gentamicin sulfate, 25 mM HEPES (Sigma, St. Louis, Missouri), and 50 μM β-mercaptoethanol (Amersham Pharmacia Biotech), pH 7.2. For both organs, the cells were counted using an optical microscope with a Neubauer chamber (66).

Cells isolated from the brain and spleen were plated in an approximate amount of 1 × 10^6^. Then 10 μL per well of the cocktail of monoclonal anti-phenotypic marker antibodies conjugated with the fluorochromes FITC, PercP-cy5.5, PE-Cy7, APC, AmCyan, and biotin (the latter was bound to streptavidin Pacific Blue later) were added. The cells were incubated at 4°C for 30 min in the dark, washed with 100 μL per well of PBS-BSA-NaN3, and centrifuged at 1,200 rpm for 10 min. The supernatant was discarded and then the washing procedure was repeated. Subsequently, the biotin-labeled cells were incubated again (for 30 min) with streptavidin-Pacific Blue and washed another two times as described. Finally, they were resuspended in 200 μL of fixative solution (0.5% formaldehyde in PBS1X) and kept at 4°C in the dark until the next day. The antibodies used were anti-CD11b (FITC); anti-F4/80 (PE-Cy7), anti-CD45.2 (biotin + streptavidin Pacific Blue), and cell viability markers (AmCyan). Reading was performed using the FACS Fortessa (Beckton Dickinson, Mountain View, California), and analyses conducted using the FlowJo program (Tree Star Inc).

### Primer design and relative quantification by RT-qPCR

*Primer* design was performed as described by (52), when necessary. Table 1 describes the sequences (5′ → 3′) of the target genes used in this study (52, 67–71). For RT-qPCR analyses, total striatum RNA was extracted as described by (52). RNA concentration and purity were investigated with a DeNovix DS-11 spectrophotometer (Delaware, United States). RNA integrity was visualized on a 1% agarose gel, stained with GelRed (Biotium, California, United States). Reverse transcription was performed with oligo primers (Dt20; Prodimol Biotecnologia, Belo Horizonte, Brazil), dNTP mix (10 mM), Reaction Buffer 5X (Thermo Fisher Scientific, São Paulo, Brazil), and M-MLV Reverse Transcriptase (Promega Wisconsin, United States), according to the manufacturer’s guidelines. Levels of gene transcription were measured using the CFX 96TM Real-Time system thermocycler (BioRad, California, United States). The RT-qPCR reactions for each gene were performed using 10 μL GoTaq® RT-qPCR Sybr (Promega, Wisconsin, United States), 1 μL cDNA (10 ng/μL), 0.4 μL of sense and antisense primer solution (10 pM), and 8.2 μL Invitrogen RT-PCR Grade Water (Thermo Fisher Scientific, Massachusetts, United States) (52). In all reactions, a negative control without cDNA template (NTC) was used, and the final reaction volume was kept at 20 μL. qPCR amplification was performed without the extension step (95°C for 3 min, followed by 40 cycles of 95°C for 3 s and 60°C for 20 s). Fluorescence levels were measured at the end of each cycle (56). The relative quantifications were calculated by the delta–delta Ct method (72). The normalizing genes used were Glyceraldehyde-3-Phosphate Dehydrogenase (*Gapdh*) and Peptidylprolyl isomerase A (*Ppia*) (71, 73). The stability of these reference genes was confirmed using the geNorm software used to evaluate multiple internal control genes (73).

**Table 1:** RT-qPCR Primers sequences (5′ → 3′).

*Gene*	Forward	Reverse	Amplicon length	Reference
*Lrrk2*	TTCCCCACCAATGAAAACAT	AAGGCTGCGTTCTCAGGATA	146	*This Study*
*Nfat*	CAGTGTGACCGAAGATACCTGG	TCGAGACTTGATAGGGACCCC	130	(67)
*Trl4*	AGTAGCACTGACACCTTCCTT	GCCTTAGCCTCTTCTCCTTCA	105	(68)
*Drd1*	GAGTCGGGGAGTGGTCT	CAATCTCAGTCACTTTTCGGGG	105	(52)
*Drd2*	GCCAACCTGAAGACACCACTCA	CTTGACAG CATCTCCATTTCCAG	158	(52)
*Il6*	CTCTGGGAAATCGTGGAAATG	AAGTGCATCATCGTTGTTCATACA	75	(69)
*Il1β*	CACTCATTGTGGCTGTGGAGAA	CCACGGGAAAGACACAGGTAG	53	(69)
*Il10*	GCTCTACTGACTGGCATGAG	CGCAGCTCTTAGGAGCATGTG	105	(70)
*iNOS*	AGCACTTTGGGTGACCACCAGGA	AGCTAAGTATTAGAGCGGCGGCA	53	(70)
*Gapdh*	AGGAGCGAGACCCCACTAAC	GTGGTTCACACCCATCACAA	179	(71)
*Ppia*	AATGCTGGACCAAACACAAA	CCTTCTTTCACCTTCCCAAA	101	(71)

### Statistical analyses

The data were evaluated for distribution with the Shapiro–Wilk normality test. They were expressed as mean ± SEM. Two-way ANOVA, followed by the *post hoc* Tukey test, was employed in the analysis of body weight at T1 and T2, adiposity index, ethanol consumption, behavioral tests, percentage of labeled cells in flow cytometry, and relative quantification of transcripts of selected genes in the striatum. The ANOVA data are represented as [F (between-group df, within-group df) = F-statistic, value of *p*]. The *Mann–Whitney* Test was used to compare the preference for ethanol with the hypothetical value of 50.1%. *Spearman* correlation with simple linear regression was used to study the relationship between the transcriptional levels of *Nfat* and *Lrrk2*. All analyses were conducted in the *GraphPad Prism* statistical package, version 9.0.2 (GraphPad Software, Inc. San Diego, United States). The significance level was *p* < 0.05 and it was indicated with an asterisk (*).

## Results

### Consumption of the HSB diet affected body weight and adiposity index and its withdrawal affected consumption and preference for ethanol

**Figure 2:**
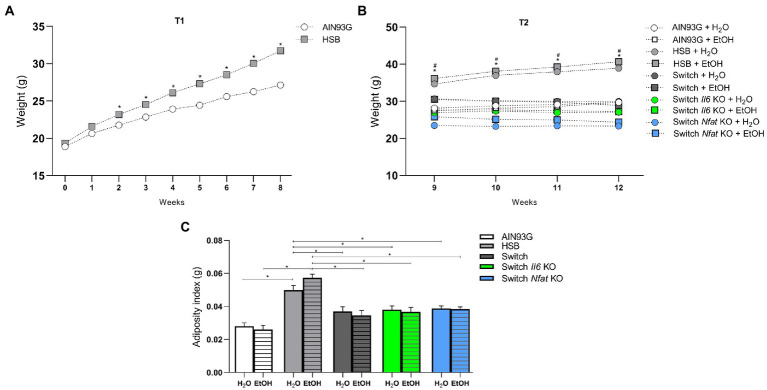
Body weight and adiposity index. **(A)** T1 body weight (g). **(B)** T2 body weight (g). **(C)** Adiposity index (g). Results are expressed as mean ± SEM. Analyses were performed with two-way ANOVA followed by *post hoc* Tukey. In **(A)** ^*^p < 0.05 for AIN93G vs. HSB. In **(B)**, ^*^p < 0.05 for HSB + H_2_O and HSB + EtOH vs. AIN93G + H_2_O, AIN93G + EtOH, Switch + H_2_O, Switch + EtOH, Switch *II6* KO + H_2_O, Switch *Il6* KO + EtOH, Switch *Nfat* KO + H_2_O and Switch *Nfat* KO and ^#^*p* < 0.05 for Switch + H_2_O vs. Switch *Nfat* KO+ H_2_O. Asterisks (^*^) and (^#^) represent *post hoc* test differences between groups.

Two-way ANOVA showed that body weight at T1 was significantly affected by diet [*F* (1, 89) = 9.087, *p* = 0.0034], by period of ingestion [*F* (2,338,208.1) = 228.9, *p* < 0.0001] and by the interaction of these factors [*F* (8, 712) = 9.110, *p* < 0.0001]. At the end of T2, the treatment carried out [*F* (9, 81) = 20.73, *p* < 0.0001], the duration of the experiment [*F* (1,901, 154.0) = 13.44, *p* < 0.0001] and the interaction between these aspects [*F* (24, 243) = 11.03, *p* < 0.0001] impacted on the weight gain of mice. The *post hoc* test revealed that the animals that ingested the HSB diet throughout the experiment had significantly higher body weight (*p* < 0.05) contrasted to the other groups, that ingested only the AIN93G diet or that at T2 the HSB diet was switched to the AIN93G formulation (Figures 2A,B). Also, at T2, the animals in the Switch + H_2_O group had higher body weight (*p* < 0.05) compared to those in the Switch *Nfat* KO + H_2_O group (Figure 2B).

In addition, the two-way ANOVA showed that the adiposity index, which shows the perigonadal fat accumulation in the animals, was influenced by diet [*F* (4, 81) = 30.40, *p* < 0.0001] and not by ethanol [*F* (1, 81) = 0.02760, *p* = 0.8685], and the *post hoc* test indicated that specimens from the HSB group (HSB + H_2_O and HSB + EtOH) had a higher adiposity index (*p* < 0.05) contrasted to animals from the groups AIN93G and Switch (AIN93G + H_2_O, AIN93G + EtOH, Switch + H_2_O, Switch + EtOH, Switch *Il6* KO + H_2_O, Switch *Il6* KO + EtOH, Switch *Nfat* KO + H_2_O, and Switch *Nfat* KO + EtOH; Figure 2C).

**Figure 3:**
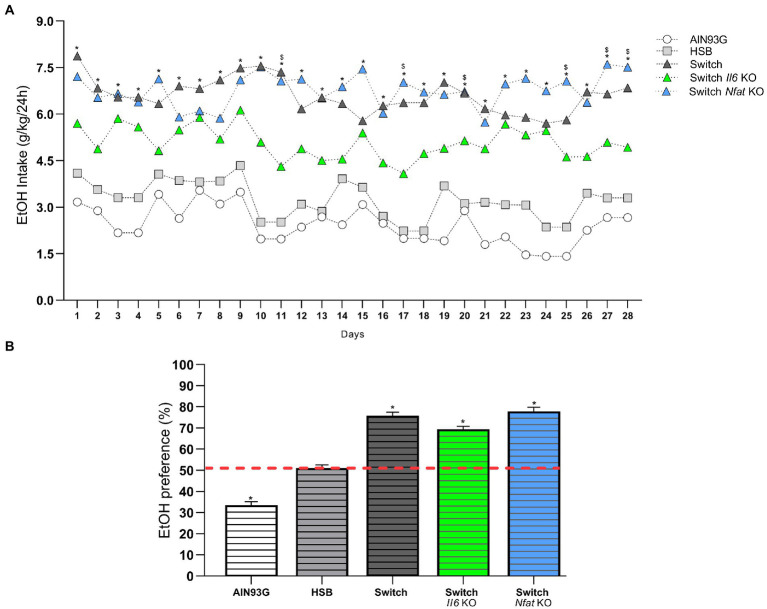
Daily intake and ethanol preference. **(A)** Ethanol intake is expressed as the ratio between daily consumption and body weight (g/Kg/24 h). **(B)** Ethanol preference is determined by (%) consumed in relation to total fluid intake. Results are expressed as mean ± SEM. Analyses were performed with two-way ANOVA followed by *post hoc* Tukey test in **(A)** and by Mann–Whitney test versus the hypothetical value of 50.1% in **(B)**. In **(A)** ^*^p < 0.05 for Switch + EtOH, Switch *Il6* KO + EtOH and Switch *Nfat* KO + EtOH vs. HSB + EtOH and AIN93G vs. EtOH and for Switch *Nfat* KO + EtOH vs. Switch *Il6* KO + EtOH on days 11, 17, 20, 25, 27, and 28. In **(B)** ^*^p < 0.05 for the hypothetical value of 50.1% vs. AIN93G + EtOH, Switch + EtOH, Switch *Il6* KO + EtOH and Switch *Nfat* KO + EtOH. Asterisks (^*^) represent *post hoc* test differences between groups.

Regarding the daily consumption of ethanol, the two-way ANOVA showed that the type of diet [*F* (4, 41) = 69.56, *p* < 0.0001] and the period of consumption of the drink [*F* (12.18, 499.5) = 3.559, *p* < 0.0001] significantly affected the animals. After switching from the HSB diet to the AIN93G diet at the end of T1, the *post hoc* test indicated that specimens from the Switch + EtOH, Switch *Il6* + EtOH, and Switch *Nfat* KO + EtOH groups consumed alcohol similarly (*p* > 0.05), except on days 11, 17, 20, 25, 27, and 28, in which the mice in the Switch *Nfat* KO + EtOH group ingested a greater amount of the drug than the animals in the Switch *Il6* KO + EtOH group. All groups with diet change (Switch + EtOH, Switch *Il6* + EtOH and Switch *Nfat* KO + EtOH) consumed significantly higher amounts of the substance (*p* < 0.05) compared to the animals in the AIN93G + EtOH and HSB + EtOH, which did not differ from each other (Figure 3A). The preference for ethanol over water was also greater (*p* < 0.0001) among specimens from the Switch + EtOH, Switch *II6* + EtOH and Switch *Nfat* KO + EtOH groups. Meanwhile, mice from the AIN93G + EtOH group showed no preference for the beverage (*p* < 0.0001) and those from the HSB + EtOH group did not express a significant result (*p* = 0.1138) in relation to the hypothetical value of 50.1% (Figure 3B).

### The consumption of the HSB diet and ethanol affected the animal behavior

**Figure 4:**
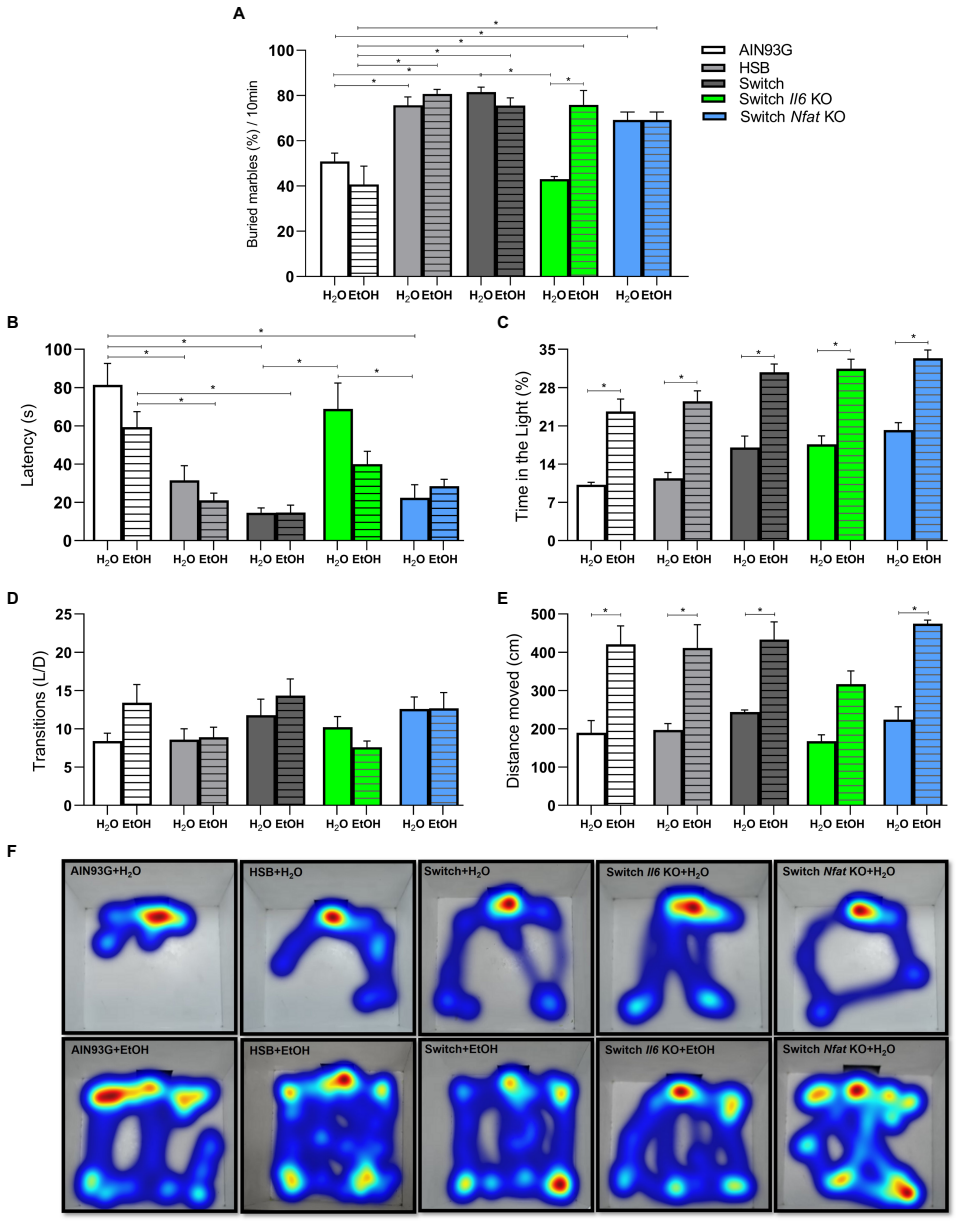
Behavioral analyses. Impulsive-like behavior was evaluated in the Marble burying test by **(A)** % of buried marbles. Anxiety-like behavior was evaluated in the light/dark box test by **(B)** Latency (s), **(C)** Time on the light (%), **(D)** Transitions number, **(E,F)** Distance moved **(CM)**. Results are expressed as mean ± SEM. Analyses were performed with two-way ANOVA followed by *post hoc* Tukey test. In **(A)** ^*^p < 0.05 for AIN93G + H_2_O vs. HSB + H_2_O and Switch + H_2_O, for AIN93G + EtOH vs. HSB + EtOH, Switch + EtOH and Switch *Il6* KO + EtOH and for Switch *Il6* KO + H_2_O vs. Switch + EtOH and Switch *Il6* KO + EtOH. In **(B)** ^*^p < 0.05 for AIN93 + H_2_O vs. HSB + H_2_O, Switch + H_2_O, and Switch *Nfat* KO + H_2_O, for AIN93G + EtOH vs. HSB + EtOH and Switch + EtOH and for Switch *Il6* + H_2_O vs. Switch + H_2_O and Switch *Nfat* + H_2_O. In **(C,E)** ^*^p < 0.05 for AIN93 + H_2_O vs. AIN93G + EtOH, for HSB + H_2_O vs. HSB + EtOH, for Switch + H_2_O vs. Switch + EtOH, for Switch *II6* KO + H_2_O vs. Switch *Il6* KO + EtOH and for Switch *Nfat* KO + H_2_O vs. Switch *Nfat* KO+ EtOH. Asterisks (^*^) represent *post hoc* test differences between groups.

Two-way ANOVA [*F* (3, 50) = 8.513, *p* = 0.0001] showed that the Marble Burying test was influenced mainly by the interaction between dietary treatment and beverage consumption. This test is used to assess impulsivity, typical characteristic of obsessive–compulsive and anxious behavior in mice (56, 57). In this sense, the *post hoc* test indicated that the animals in the AIN93G group (AIN93G + H_2_O and AIN93G + EtOH) expressed low impulsivity compared to those in the HSB groups (HSB + H_2_O and HSB + EtOH), Switch (Switch + H_2_O and Switch + EtOH), Switch *Il6* KO + EtOH and Switch *Nfat* KO (Switch *Nfat* KO + H_2_O and Switch *Nfat* KO + EtOH; Figure 4A). Interestingly, mice from the Switch *Il6* KO + H_2_O group showed low impulsivity contrasted to those from the Switch Il6 KO + EtOH group, while no differences were observed in relation to the specimens from the Switch *Nfat* KO group (Figure 4A).

The light/dark box test is traditionally applied in assessing anxious-like behavior in animal models, using metrics such as latency, time spent in the light compartment, number of transitions, and distance traveled within the box (46, 56). In this context, two-way ANOVA revealed that diet and ethanol affected both latency [diet: *F* (4, 67) = 20.68, *p* < 0.0001; ethanol: *F* (1, 67) = 5.065, *p* = 0.0277] and time spent in the light compartment [diet: *F* (4, 73) = 9.551, *p* < 0.0001; ethanol: *F* (1, 73) = 94.23, *p* < 0.0001], while alcohol alone [*F* (1, 1, 64) = 75.28, *p* < 0.0001] influenced the results related to the distance traveled inside the bright part of the box.

For latency, the *post hoc* test indicated that animals from the HSB (HSB + H_2_O and HSB + EtOH), Switch (Switch + H_2_O and Switch + EtOH) and Switch *Nfat* KO (Switch *Nfat* KO + H_2_O and Switch *Nfat* KO + EtOH) started exploring the bright part of the compartment in a shorter time (*p* < 0.05) than specimens from the AIN93G group (AIN93G + H_2_O and AIN93G + EtOH), demonstrating an anxiolytic effect of the treatment for these animals (Figure 4A). Meanwhile, those in the Switch *Il6* KO + H_2_O group exhibited a longer latency time contrasted to those in the Switch + H_2_O and Switch *Nfat* KO + H_2_O groups (Figure 4B). No differences were observed in relation to the Switch *Il6* KO + EtOH group (Figure 4B). Mice from the +EtOH groups (AIN93G + EtOH, HSB + EtOH, Switch + EtOH, Switch *Il6* KO + EtOH, and Switch *Nfat* KO + EtOH) spent more time (*p* < 0.05) in the light compartment of the box than those of +H_2_O groups (AIN93G + H_2_O, AIN93G + H_2_O, Switch + H_2_O, Switch *Il6* KO + H_2_O, and Switch *Nfat* KO + H_2_O; Figure 4C). These data again confirm the anxiolytic effect of the experimental treatments. No significant differences were noticed regarding the number of light/dark transitions (Figure 4D).

Regarding the distance covered in the bright part of the box, the *post hoc* test showed that the animals in the +EtOH groups (AIN93G + EtOH, HSB + EtOH, Switch + EtOH, Switch *Il6* KO + EtOH, and Switch *Nfat* KO + EtOH) covered a greater distance (*p* < 0.05) and hence explored the environment more than the +H_2_O groups (AIN93G + H_2_O, AIN93G + H_2_O, Switch + H_2_O, Switch *Il6* KO + H_2_O, and Switch *Nfat* KO + H_2_O; Figure 4E). This result is pictured by the Heatmaps presented in Figure 4F, in which the greater exploitation of the area by the animals of the +EtOH groups becomes evident.

### Dietary treatment and ethanol consumption affected the percentage of inflammatory cells in the brain and spleen

**Figure 5:**
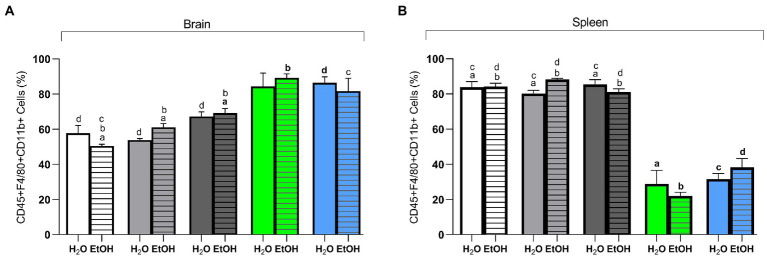
Flow cytometry of brain and spleen cells. **(A)** CD45 + F4/80 + CD11b + (%) in the brain. **(B)** CD45 + F4/80 + CD11b + (%) in the spleen. Results are expressed as mean ± SEM. Analyses were performed with two-way ANOVA followed by *post hoc* Tukey test. In **(A)** *p* < 0.05 for **(a)** AIN93G + EtOH vs. HSB + EtOH and Switch + EtOH, for **(b)** HSB + EtOH vs. Switch + EtOH and Switch *Il6* KO + EtOH, for **(c)** AIN93G + EtOH vs. Switch + EtOH and **(d)** for AIN93G + H_2_O vs. HSB + H_2_O, Switch + H_2_O and Switch *Nfat* KO + H_2_O. In **(B)** *p* < 0.05 for **(a)** Switch *II6* + H_2_O vs. AIN93G + H_2_O, HSB + H_2_O and Switch + H_2_O, for **(b)** Switch *II6* + EtOH vs. AIN93G + EtOH, HSB + EtOH, and Switch + EtOH, **(c)** for Switch *Nfat* + H_2_O vs. AIN93G + H_2_O, HSB + H_2_O, and Switch + H_2_O and **(d)** for Switch *Nfat* + EtOH vs. AIN93G + EtOH, HSB + EtOH, and Switch + EtOH.

In the brain, two-way ANOVA revealed that diet is the main factor for changes in the percentages of CD45 + F4/80 + CD11b + cells [*F* (4, 27) = 36.43, *p* < 0.0001]. The *post hoc* test showed that the animals in the Switch *Il6* KO + EtOH group had a higher (*p* < 0.05) percentage of CD45 + F4/80 + CD11b + cells than the animals in the AIN93G + EtOH, HSB + EtOH, and Switch + EtOH groups. Likewise, it was indicated that the animals in the Switch + EtOH group have a higher percentage of this marker in their cells than those in the AIN93G + EtOH and HSB + EtOH groups (Figure 5A). The Switch *Nfat* KO + EtOH group has a higher percentage when compared to the AIN93G + EtOH group (Figure 5A). For the same marker, the Switch *Nfat* KO + H_2_O group has higher amounts than the AIN93G + H_2_O, HSB + H_2_O, Switch + H_2_O groups (Figure 5A). In the spleen, diet [*F* (4, 27) = 108.0, *p* < 0.0001] was responsible for the differences in cells labeled with CD45 + F4/80 + CD11b. The *post hoc* test confirmed that the AIN93G, HSB, and Switch (AIN93G + H_2_O, AIN93G + EtOH, HSB + H_2_O, HSB + EtOH, Switch + H_2_O, and Switch + EtOH) groups have higher percentages (*p* < 0.05) of CD45 + F4/80 + CD11b + cells when compared to *Il6* and *Nfat* KO animals (Switch *Il6* + H_2_O, Switch *Il6* + EtOH, Switch *Nfat* + H_2_O, and Switch *Nfat* + EtOH; Figure 5B).

### Ethanol consumption affected the transcription of *Trl4*, *Lrrk2*, *Nfat*, and cytokines in the striatum

**Figure 6:**
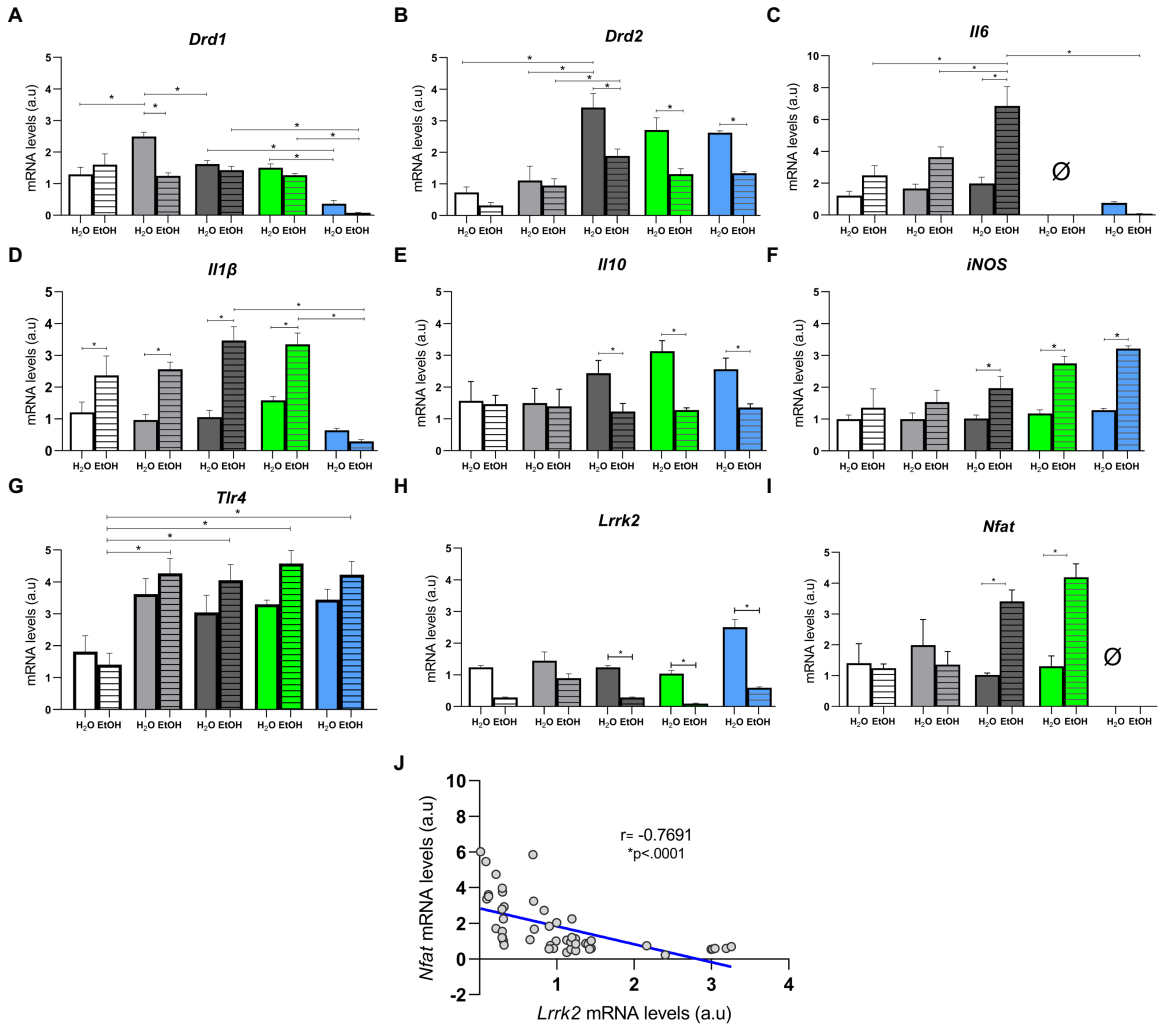
Relative mRNA quantification in the striatum. Relative mRNA levels in **(A)** *Drd1*, **(B)** *Drd2*, **(C)** *Il6*, **(D)** *Il1β*, **(E)** *Il10*, **(F)** *iNOS*, **(G)** *Tlr4, **(H)** Lrrk2*, and **(I)** *Nfat*. Results are expressed as mean ± SEM. Analyses were performed with two-way ANOVA followed by *post hoc* Tukey test. **(J)** Spearman correlation between mRNA levels of the *Lrrk2* and *Nfat* genes. In **(A)** ^*^p < 0.05 for AIN93G + H_2_O vs. HSB + H_2_O, for HSB + H_2_O vs. HSB + EtOH and Switch + H_2_O, for Switch + H_2_O vs. Switch *Nfat* KO + H_2_O, for Switch + EtOH vs. Switch *Nfat* KO + EtOH, for Switch *Il6* KO + H_2_O vs. Switch *Nfat* KO + H2O and for Switch *Il6* KO + EtOH vs. Switch *Nfat* KO + EtOH. In **(B)** ^*^p < 0.05 for Switch + H_2_O vs. AIN93G + H_2_O, HSB + H_2_O and Switch + EtOH, for Switch *Il6* KO + H_2_O vs. Switch *Il6* KO + EtOH and for Switch *Nfat* KO + H_2_O vs. Switch *Nfat* KO + EtOH. In **(C)** ^*^p < 0.05 for Switch + EtOH vs. AIN93G + EtOH, HSB + EtOH, Switch + H_2_O and Switch *Nfat* KO + EtOH. In **(D)** ^*^p < 0.05 for AIN93G + H_2_O vs. AIN93G + EtOH, for HSB + H_2_O vs. HSB + EtOH, for Switch + EtOH vs. Switch + H_2_O and for Switch *Nfat* KO + EtOH, for Switch *Il6* KO + EtOH vs. Switch *Il6* KO + H_2_O and Switch *Nfat* KO + EtOH. In **(E,F,H)** ^*^p < 0.05 for Switch + H_2_O vs. Switch + EtOH, for Switch *Il6* KO + H_2_O vs. Switch *Il6* KO + EtOH and for Switch *Nfat* KO + H_2_O vs. Switch *Nfat* KO + EtOH. In G ^*^p < 0.05 for AIN93G + EtOH vs. HSB + EtOH, Switch + EtOH, Switch *II6* KO + EtOH and Switch *Nfat* KO + EtOH. In **(I)** ^*^p < 0.05 for Switch + H_2_O vs. Switch + EtOH and for Switch *Il6* KO + H_2_O vs. Switch *Il6* KO + EtOH.

Transcriptional regulation of key genes and cytokines related to ethanol consumption and dependence were affected in the different experimental groups of the present study (57). Two-way ANOVA showed that alcohol is the only factor responsible for differences in the transcriptional regulation of genes *Nfat* [*F* (1, 47) = 11.67, *p* = 0.0013], *Il1β* [*F* (1, 46) = 42.02, *p* < 0.0001], *Il10* [*F* (1,44) = 13.40, *p* = 0.0007], and *iNOS* [*F* (1,46) = 35.00, *p* < 0.0001]. Whereas *Drd1* [*F* (4, 44) = 5.476, *p* = 0.0011], *Drd2* [*F* (4, 42) = 2. 642, *p* = 0.0469], *Il6* [*F* (2, 27) = 3.788, *p* = 0.0355], *Tlr4* [*F* (4, 39) = 1.141, *p* = 0.3516], and *Lrrk2* [*F* (4, 46) = 11.68, *p* < 0.0001] were responsible for the observed differences in the interaction between diet and ethanol consumption. We evaluated the transcription of dopamine receptors *Drd1* and *Drd2* in the striatum. *Post hoc* analysis concluded that *Drd1* was upregulated in HSB + H_2_O when compared to AIN93G + H_2_O, HSB + EtOH, and Switch + EtOH, while it was downregulated on Switch *Nfat* KO + H_2_O and + EtOH compared to Switch *II6* KO + H_2_O and + EtOH, and Switch + H_2_O and + EtOH, respectively (Figure 6A). *Drd2* was upregulated in Switch + H_2_O when compared to AIN93G + H_2_O, HSB + H_2_O and Switch + EtOH; Switch + EtOH versus HSB + EtOH, Switch *Il6* KO + H_2_O versus Switch *Il6* KO + EtOH, and Switch *Nfat* KO + H_2_O versus Switch *Nfat* KO + EtOH (Figure 6B).

As for inflammatory cytokines (*Il6* and *Il1β*) and anti-inflammatory (*Il10*), *post hoc* analysis revealed that *Il-6* was upregulated (*p* < 0.05) in the Switch + EtOH group contrasted to animals in the HSB + EtOH, AIN93G + EtOH, and Switch *Nfat* KO + EtOH groups (Figure 6C). *Il-1β* was downregulated in HSB + H_2_O, Switch + H_2_O and Switch *Il6* KO + H_2_O animals compared to HSB + EtOH, Switch + EtOH, and Switch *Il6* KO + EtOH animals, respectively, and Switch *Nfat* KO + EtOH compared to Switch + ETOH and Switch *Il6* KO + EtOH (Figure 6D). *Il-10* was upregulated in animals from the Switch + H_2_O, Switch *Il6* KO + H_2_O, and Switch *Nfat* KO + H_2_O groups compared to animals from the Switch + EtOH, Switch *Il6* KO + EtOH, and Switch *Nfat* KO + EtOH (Figure 6E). The *iNOS* gene showed downregulation in the Switch + H_2_O, Switch *Il6* KO + H_2_O, and Switch *Nfat* KO + H_2_O groups contrasted to the Switch + EtOH, Switch *Il6* KO + EtOH, and Switch *Nfat* KO + EtOH groups, respectively (Figure 6F). The *Tlr4* gene was downregulated in the AIN93G + EtOH group when compared to other groups that had access to alcohol (Figure 6G). The *Lrrk2* gene, which was recently associated with loss of control and preference for ethanol by our research group, showed downregulation in groups that consumed high ethanol (Switch + EtOH, Switch *Il6* KO + EtOH, and Switch *Nfat* KO + EtOH; Figure 6H). Conversely, the *Nfat* gene was upregulated in these groups (Figure 6I). Interestingly, Spearman’s correlation demonstrated a negative relationship between the *Lrrk2* and *Nfat* genes (*r* = 0.7691, regression equation −1.007*X + 2.840, *p* < 0.0001; Figure 6J).

## Discussion

Intending to evaluate the transcriptional regulation of the *Lrrk2* gene in association with genes related to the immune system and its implications on the behavior and ethanol preference in C57BL/6 mice, we reproduced a model of chronic consumption of HSB diet and free choice of alcohol, as detailed by (52, 53). Here, we also conducted the model on two KO animals’ lines, *Il6* and *Nfat*. In this previously mentioned model, it was observed that, after feeding the mice for 8 weeks with the HSB diet and its subsequent switch to the AIN93G diet, it was verified in the animal’s high ethanol consumption and preference (52, 53). Additionally, animals that consumed only the HSB diet developed obesity-like characteristics (54, 56). We reproduced these results as seen in Figures 2, 3 and observed that the absence of Il6 and Nfat genes does not significantly alter the ethanol preference and consumption in animals over time. Indeed, we had already demonstrated that the results observed in this model are linked to instability in the mesocorticolimbic dopaminergic pathway due to alterations in the transcriptional regulation of dopamine receptors (*Drd1*/*Drd2*) and here, in addition to reproducing these results, we demonstrate that in the absence of *Il6* or *Nfat*, the transcription profile of these genes remains in high consumption and preference for ethanol (52, 53).

Concerning behavior, the Marble Burying test showed greater impulsivity in animals that had access to the HSB diet in T1, being comparable to the obsessive–compulsive disorder seen in humans (59, 74, 75). This result is consistent with studies in which hypercaloric diets were associated with binge eating (56, 76, 77). Thus, this allows us to suggest that the HSB diet consumption can trigger an obsessive–compulsive-like disorder in mice and that our model can be applied in studies related to bariatric surgery in humans, mainly regarding the relationship of this procedure with alcohol consumption. Studies show that in the 2-year postoperative follow-up to bariatric surgery, about 8.3% of patients develop disorders related to alcohol use (78–80). In general, it is believed that there is a transfer from compulsive eating to ethanol high consumption and loss of inhibitory control in individuals after bariatric surgery, which potentiates preference for the substance, as observed in the Switch groups (Switch + EtOH, Switch *Il6* KO + EtOH, and Switch *Nfat* KO + EtOH) (81–83).

Ethanol use is associated with many other behavioral changes (84). In the present study, we observed an anxiolytic effect associated with ethanol chronic consumption, evidenced by low latency, high permanence time, greater distance moved, and exploration of the light compartment in the light/dark box test in animals that were in groups associated with the paradigm free choice for ethanol, and it is important to highlight that this behavior was observed regardless of the amount ingested. This finding is corroborated by several animal model studies that show the anxiolytic effect of the consumption of this drug (10, 61, 85, 86). Considering that anxiety is a defense strategy in mice, as it is associated with an aversion to open and lit places that make them more visible to potential predators, the reduction in that behavior harms them (62, 87). In this context, this effect is also deleterious for humans since it is a positive reinforcement in situations of high anxiety and depressive symptoms, which may be one of the key factors for the development and maintenance of drug dependence (88, 89). Therefore, several strategies are investigated to block the ethanol anxiolytic effects; for example, Correia et al. (90) used catalase activity-blocking drugs that inhibited ethanol metabolism in the brain and they succeeded in mitigating the alcohol anxiolytic effects on animals (90). Thus, the authors concluded that psychopharmacological strategies that block the ethanol action in the brain can be used to reduce the dependence effects of this drug (90). Given this, the model developed here can be efficient in the study of ethanol-related neurobiological and behavioral changes, especially for studies aimed at blocking the alcohol anxiolytic effects in animal models.

Considering yet the behavioral tests, we observed that changing the HSB to AIN93G diet had a different impact on the behavior of the animals in the Switch *Il6* KO + EtOH group. These animals showed higher impulsivity compared to the H_2_O animals, which in turn had a lower latency in the light/dark box than the animals in the Switch + H_2_O and Switch *Nfat* KO + H_2_O groups. Although these results do not have direct implications concerning ethanol consumption in *Il6* KO animals, they should be considered in relation to immune response and inflammatory molecules importance in behavioral changes, since the immune system is implicated in several psychiatric diseases development, such as depression, bipolar disorder, schizophrenia, autism, anxiety, and AUD (19, 91–93).

In general, it is known that ethanol exposure activates neuroimmune signaling, causing a highly inflammatory environment in the brain, in which microglial cells, macrophages and monocytes infiltrates increase pro-inflammatory markers transcription (IL1β, IL6), reduce anti-inflammatory markers (IL10) and increase levels of nitric oxide synthase (iNOS) (25, 94–97). In this context, in an extensive review of the topic, Montesinos et al. describe ways in which ethanol regulates the transcription of neuroimmune and microglial genes and, in particular, mention the activation of toll-like receptors four present in immune cells and neurons by bacterial components that activate the inflammatory response (18). In fact, in previous studies, our research group described changes in the abundance and structure of gut microbiota and proposed that the increase in bacterial translocation to the bloodstream would allow those bacterial components to activate the cerebral inflammatory response (53, 96). In the present study, in addition to demonstrating a possible change in *Tlr4* transcription in animals that intaken alcohol, we also observed an increase in macrophages (CD45 + F4/80 + CD11b+) in the brain of animals in the Switch group (Switch + EtOH, Switch *Il6* KO + EtOH, and Switch *Nfat* KO + EtOH) (98, 99). Functionally, CD11b regulates the adhesion and migration of leukocytes for the mediation of the inflammatory response (100–102). Therefore, the potential for neuroinflammation to be induced by the gut microbiota in these animals should not be overlooked. In the spleen, there was a decrease in macrophages (CD45 + F4/80 + CD11b+) of the *Il6* KO and *Nfat* KO animals, showing that these genes are important for the maintenance of these cells in the organ in view of the treatments given to the animals be capable of affecting and modifying pathways associated with systemic inflammation processes in their organisms (103, 104). Specifically in the striatum, brain region sensitive to ethanol, activation of neuroimmune signaling is observed with drug consumption. By exposing mice to ethanol for 8 weeks, Asatryan et al. observed increased neurodegeneration and transcription of pro-inflammatory mediators in striatum (94). This supports our findings since we observed in the striatum an upregulation of genes related to pro-inflammatory cytokines *Il6* and *Il1β* and a downregulation of *Il10* in mice with ethanol high consumption (Switch + EtOH). Interestingly, we observed iNOS upregulation in animals from the Switch + EtOH, Switch *Il6* KO + EtOH, and Switch *Nfat* KO + EtOH groups, which is logical because of the inverse relationship with the transcript of *Il10* since this interleukin is known to suppress iNOS induction (105). Thus, the *Il10* low levels in animals with ethanol high intake and preference may be contributing to the iNOS increase whose expression is the result of a localized or diffuse inflammatory response (106, 107). In parallel with the results described, we found a *Lrrk2* downregulation and a *Nfat* upregulation in these groups, which makes sense, considering its NFAT inhibitory role (45, 48). So, with a lower *Lrrk2* transcription, there are higher levels of NFAT, which can trigger an increase in inflammatory cytokines depending on that molecule and which play a key role in the inflammation process. In fact, we found a negative correlation between these genes that suggests the possibility that low *Lrrk2* transcription could increase neuroinflammation and drive in part the ethanol dependence phenotype cannot be disregarded. This is confirmed without the *Nfat* gene, where there are inflammatory cytokines with low transcription, showing its importance in inducing these in the striatum in the face of ethanol intake and a close relationship with *Lrrk2*.

In this context, our research group identified relations of the *Lrrk2* gene, until then associated with Parkinson’s and inflammatory bowel diseases, in preference behavior and loss of control by alcohol in mice, Zebrafish, and humans (31, 32, 36, 108–110). This gene is involved in neural processes, such as the reuptake of synaptic components and synaptic plasticity, and in several signaling pathways by performing GTPase and kinase functions, which influence processes such as cell proliferation and differentiation, apoptosis, inflammation, and immune response (50, 111–116). Evidence in humans and mice corroborates with the results of this study regarding the differential regulation with *Lrrk2* downregulation to alcohol intake. In this scenario, in humans, *Lrrk2* downregulation was observed in the post-mortem brain of patients with AUD, and mice, it was observed that alcohol decreases the activity of LRRK2 kinase in the striatum and that deletion of the gene in striatal neurons that express D1 promotes the binge drinking by D1 receptor signaling and function (31, 36). Despite this, some studies have shown the opposite results. In a model of loss of control by ethanol in mice, a *Lrrk2* hyperregulation was observed in the striatum of animals that showed a preference for the substance even after the adverse stimulus and the inhibition of the kinase function of this gene in Zebrafish reduced the ethanol preference (31, 109). The conflicting results found in these studies are difficult to compare concerning this study, because of the existing differences regarding the treatments used, experimental models, ethanol time of exposure, and concentration. Hence, even with discrepant findings, our results allow us to propose that alterations in the transcriptional regulation profile of this gene play a key role in ethanol high consumption and preference and with the immune response in the striatum.

In summary, the results of the present study demonstrate how the animal model of HSB diet consumption and ethanol ingestion developed by our group is reproducible and can be efficient in the study of alterations related to AUD. With this model, we also showed that ethanol consumption affected (I) the behavior of the animals, (II) neuroinflammation, and (III) the regulation of the transcription of genes *Tlr4*, *Lrrk2*, *Nfat,* and cytokines in the striatum, where there is an increase of inflammatory and reduction of anti-inflammatory cytokines. Thus, the data obtained in the study contribute to a better understanding of the factors associated with the neurobiology of AUD and, consequently, to the elucidation of new therapeutic targets for this disease.

## Data availability statement

The original contributions presented in the study are included in the article/supplementary material, further inquiries can be directed to the corresponding author.

## Ethics statement

The animal study was reviewed and approved by the ethics committee of the university (CEUA-UFMG; protocol number: 73/2021). Every effort was made to ensure animal welfare.

## Author contributions

RM conducted the animal model, performed the molecular and behavioral analyses, contributed to Leukocyte extraction, and wrote the paper. MG performed the Leukocyte extraction and flow cytometry. ME contributed to the experiments and statistical analysis. TM and AF developed the HSB diet and helped to discuss the results. AB contributed to the project development and supervised all the study steps. All authors contributed to the article and approved the submitted version.

## Funding

RM received a doctoral fellowship from the Coordenação de Aperfeiçoamento de Pessoal de Nível Superior—Brazil (CAPES, Ministry of Education, Brazil) and a pos-doctoral grand by the Fundação de Amparo à Pesquisa do Estado de Minas Gerais (FAPEMIG), APQ-04517-22. This work was supported by the Fundação de Amparo à Pesquisa do Estado de Minas Gerais (FAPEMIG), APQ-01213-21 and APQ-04517-22, Pró-Reitoria de Pesquisa (PRPq), and Pós-Graduação em Genética from the Universidade Federal de Minas Gerais (UFMG), Brazil.

## Conflict of interest

The authors declare that the research was conducted in the absence of any commercial or financial relationships that could be construed as a potential conflict of interest.

## Publisher’s note

All claims expressed in this article are solely those of the authors and do not necessarily represent those of their affiliated organizations, or those of the publisher, the editors and the reviewers. Any product that may be evaluated in this article, or claim that may be made by its manufacturer, is not guaranteed or endorsed by the publisher.
